# pGALS – paediatric Gait Arms Legs and Spine: a simple examination of the musculoskeletal system

**DOI:** 10.1186/1546-0096-11-44

**Published:** 2013-11-12

**Authors:** Helen E Foster, Sharmila Jandial

**Affiliations:** 1 Musculoskeletal Research Group, Newcastle University, Newcastle upon Tyne and Great North Children’s Hospital, Newcastle upon Tyne, UK

**Keywords:** pGALS, Clinical skills, Musculoskeletal assessment, Education

## Abstract

We describe pGALS (paediatric Gait, Arms, Legs and Spine) – a simple quick musculoskeletal assessment to distinguish abnormal from normal joints in children and young people. The use of pGALS is aimed at the non-specialist in paediatric musculoskeletal medicine as a basic clinical skill to be used in conjunction with essential knowledge about red flags, normal development and awareness of patterns of musculoskeletal pathologies. pGALS has been validated in school-aged children and also in the context of acute general paediatrics to detect abnormal joints. We propose that pGALS is an important part of basic clinical skills to be acquired by all doctors who may be involved in the care of children. The learning of pGALS along with basic knowledge is a useful way to increase awareness of joint disease, facilitate early recognition of joint problems and prompt referral to specialist teams to optimise clinical outcomes. We have compiled this article as a resource that can be used by the paediatric rheumatology community to facilitate teaching.

## Review

### Introduction

Children and young people (CYP) present commonly with musculoskeletal (MSK) problems [1,2] and often present initially to clinicians who are not specialists in paediatric MSK (pMSK) medicine; such clinicians include doctors working in family medicine, emergency medicine, orthopaedics, paediatrics or adult rheumatology. Furthermore, in resource-poor countries, allied health professionals are important first points of contact for CYP with MSK problems.

The majority of causes of MSK presentations in childhood are benign, self-limiting and often trauma related; referral to specialist care is not always necessary, and in many instances reassurance alone may suffice. However, MSK symptoms can be presenting features of Juvenile Idiopathic Arthritis (JIA), and potentially life-threatening conditions such as malignancy, infection, vasculitis and non-accidental injury. Inflammatory arthritis is an associated feature of many chronic paediatric conditions such as inflammatory bowel disease, cystic fibrosis, and psoriasis. It is therefore important that all clinicians to whom CYP with MSK problems may present, have the necessary skills to effectively triage patients, and where appropriate instigate referral to specialist services. We know however, that many such doctors report lack of confidence and competence in their pMSK clinical skills [3-6], stemming from a lack of pMSK teaching at undergraduate [7] and postgraduate level [8-11]. This article describes pGALS, (paediatric Gait, Arms, Legs, Spine) which we believe is an ideal tool to be learned as a basic pMSK clinical skill and may help to facilitate effective triage of CYP with MSK presentations.

### What is pGALS?

**Figure 1:**
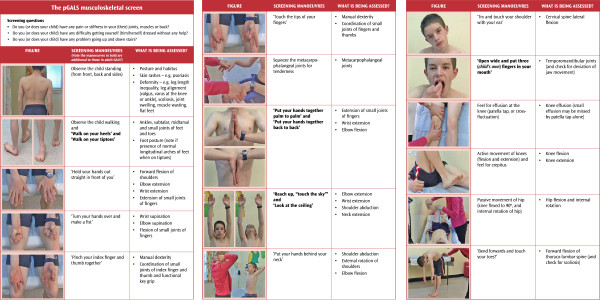
**The pGALS assessment.** Reproduced by kind permission of Arthritis Research UK (http://www.arthritisresearchuk.org) from: Foster HE, Jandial S. pGALS – A Screening Examination of the Musculoskeletal System in School-Aged Children. Reports on the Rheumatic Diseases (Series 5), Hands On 15. Arthritis Research Campaign; 2008 June.

pGALS is an evidence-based approach to basic pMSK assessment [12] and is aimed at the non-specialist in pMSK medicine to discern normal from abnormal. The components of pGALS (Figure 1) are essentially the same as adult GALS [13] with additional manoeuvres included because when adult GALS was originally tested in children with JIA [12], it missed significant abnormalities at the foot and ankle, wrists and temporomandibular joints. pGALS has been demonstrated to have excellent sensitivity to detect abnormality, incorporates simple manoeuvres often used in clinical practice, and is quick to do, taking an average of two minutes to complete [12]. pGALS was originally developed and validated in school aged children, with excellent practicality and acceptability [12]. Our experience shows that pGALS may be successfully performed in younger, ambulant children albeit observers need to be opportunistic pending cooperation and attention span of the child. When performed by non-specialists in pMSK medicine, in acute paediatric practice both in the UK and Malawi, pGALS has been shown to be practical and useful, with excellent acceptability by children and their parents [14-16]. Furthermore, pGALS is easily and effectively learned by medical students (Rowan, paper in preparation).

### When should pGALS be performed?

When there are indicators of potential MSK disease, a “top-to-toe” basic examination, such as pGALS, is helpful in the clinical assessment. Given the broad spectrum of MSK presentations, a low threshold for performing pGALS is advised, of particular importance in certain clinical scenarios (List 1), and when inflammatory joint disease is suspected (List 2); children with JIA may not complain overtly of pain [15] and symptoms can be vague and illocalised. We also suggest pGALS in the context of the child who is “clumsy” – a term often used by parents concerned about their child’s mobility or co-ordination. Traditionally a “clumsy child presentation” directs the observer to consider a neurological cause; however it is not unusual for children with MSK problems, including inflammatory disease, to present with abnormal gait, proneness to falling, or dexterity problems and we would recommend that the assessment includes pGALS as a minimum.

List 1 When to perform pGALS

• Unwell child with pyrexia

• Child with limp

• Delay or regression of motor milestones

• Child with chronic disease and known association with MSK presentations (such as inflammatory bowel disease)

• The ‘clumsy’ child in the absence of neurological disease

List 2 When inflammatory joint disease is suspected

• The lack of reported pain does not exclude arthritis

• There is need to probe for symptoms such as

○ gelling (e.g. stiffness after long car rides)

○ altered function (e.g. play, handwriting skills, writing, regression of milestones)

○ deterioration in behaviour (irritability, poor sleeping)

• There is need to examine all joints as often joint involvement may be ‘asymptomatic’

pGALS was developed in the context of detecting inflammatory joint disease but has been shown to be useful in identifying other joint problems (e.g. orthopaedic problems at the hip, scoliosis, hypermobility), joint involvement in mucopolysaccharidoses [Chan M, submitted], as well as other pathologies (stroke and sepsis) so findings need to be interpreted in the context of the physical examination elsewhere (e.g. chest, abdomen, neurological examinations) [14,16].

### pGALS as an integral part of clinical assessment

In order to assess a child presenting with MSK features, the observer using pGALS, requires knowledge of normal development, normal variants, patterns of pathologies at different ages and knowledge of indicators to warrant referral as part of early triage. Systemic upset and the presence of bone rather than joint pain may be features of MSK disease and are ‘red flags’ (List 3) that warrant urgent referral. In contrast to adults where the majority of the diagnosis can be elicited from the history, this is not the case in CYP, especially the young where the history is often given by the parent or carer, may be based on observations and interpretation of events made by others (such as teachers), may be rather vague with non-specific complaints (e.g. ‘my child is limping’) and young children may have difficulty in localising or describing pain in terms that adults may understand. In children, it is not uncommon to find joint involvement that has not been mentioned as part of the presenting complaint [17]; it is essential to perform all components of pGALS, followed up with more detailed physical examination as appropriate.

**List 3 RED FLAGS (to raise concern about infection, or malignancy or non-accidental injury)**

• Fever, systemic upset (malaise, weight loss, night sweats)

• Lymphadenopathy, hepatosplenomegaly

• Bone pain

• Persistent night waking

• Incongruence between history and presentation/pattern of physical findings

### Use of pGALS to distinguishing normal from abnormal

Key to appropriate interpretation of pGALS is knowledge of ranges of normal joint movement in different ethnicity and age groups, looking for asymmetry and careful examination for subtle changes. It is essential to check for verbal and non-verbal clues of discomfort, which may suggest joint pathology (such as facial expression, withdrawal of limb, or refusal to be examined further). Furthermore it is important that observers are aware of normal variants in gait, leg alignment and normal motor milestones (List 4 and Table 1) as these are a common cause of parental concern, especially in the pre-school child, and often can be allayed with explanation and reassurance. Normal variants do not cause pain and it is important not to ascribe MSK symptoms to the co-existence of a normal variant (e.g. the 3 year child with a limp and observed to have flat feet – the latter being normal at this age, but limp is not). Indicators for concern regarding normal variants are given (List 4).

List 4 Normal variants in gait patterns and leg alignment

Habitual **toe walking** is common in young children up to 3 years.

**In toeing** can be due to:

• ***Persistent femoral ante version*** (characterised by child walking with patellae and feet pointing inwards and is common between ages of 3–8 years)

• ***internal tibial torsion*** (characterized by child walking with patella facing forward and toes pointing inwards and is common from onset of walking to 3 years)

• ***metatarsus adductus*** (characterized by a flexible ‘C shaped’ lateral border of the foot and most resolve by 6 years).

**Bow legs** (genu varus) are common from birth to early toddler, often with out-toeing (maximal at approximately 1 year of age), and most resolve by 18 months.

**Knock knees** (genu valgus) are common and are often associated with in-toeing (maximal at approximately 4 years of age) and most resolve by age of 7 years.

**Flat feet** – most children have a flexible foot with normal arch on tiptoeing and resolve by 6 years.

**Crooked toes** – most resolve with weight bearing.

Normal variants: indications for referral

• Persistent changes (beyond the expected age ranges)

• Progressive/asymmetrical changes

• Short stature or dysmorphic features

• Painful changes with functional limitation

• Regression or delayed motor milestones

• Abnormal joint examination elsewhere

• Suggestion of neurological disease/developmental delay

**Table 1:** Normal major motor milestones

Sits without support	6–8 months
Creep on hands and knees	9–11 months
Cruise/or bottom shuffle	11–12 months
Walk independently	12–14 months
Climb up stairs on hands and knees	approx 15 months
Run stiffly	approx 16 months
Walk down steps (non-reciprocal)	20–24 months
Walk up steps, alternate feet	3 yrs
Hop on one foot, broad jump	4 yrs
Skipping	5 yrs
Balance on one foot 20 secs	6–7 yrs

Joint abnormalities can be subtle or difficult to appreciate in the young (such as ‘chubby’ ankles, fingers, wrists and knees). Looking for asymmetrical changes is helpful although can be falsely reassuring in the presence of symmetrical joint involvement. Muscle wasting (such as of the quadriceps or calf muscles) indicates chronicity of joint disease (knee or ankle respectively). Increased symmetrical calf muscle bulk associates with types of muscular dystrophy, and proximal myopathies may be suggested by delayed milestones such as walking (later than 18 months) or inability to jump (in the school-aged child).

Ranges of joint movement should be symmetrical and an appreciation of the ‘normal’ range of movement in childhood can be gained with increased clinical experience. Hypermobility may be generalised or limited to peripheral joints such as hands and feet, and, generally speaking, younger female children and those of non-Caucasian origin are more flexible. It is important to consider ‘non-benign’ causes of hypermobility such as Marfan’s syndrome (may be suggested by tall habitus with long thin fingers, and high arch palate), and Ehler’s Danlos syndromes (may be suggested by easy bruising, skin elasticity with poor healing after minor trauma). Conversely, lack of joint mobility, especially if symmetrical and in a child with short stature, dysmorphism or developmental delay, may suggest storage diseases or skeletal dysplasias. Asymmetrical loss of range is always significant and the absence of normal arches on tiptoe suggests a non-mobile flat foot and warrants investigation (e.g. to exclude tarsal coalition). High fixed arches and persistent toe walking may suggest neurological disease.

### The components of the pGALS assessment

Figure 1 lists the key components of pGALS and practical tips to facilitate the examination are given (List 5). Overt MSK complaints, or a positive response to any of the three pGALS screening questions (Figure 1), necessitates further probing. However, a negative response to these questions in the context of a MSK complaint does not exclude significant MSK disease, especially the very young. It is noteworthy that the pGALS screening questions may not be socio-culturally relevant (e.g. walking up and down stairs in environments without steps, or getting dressed and undressed in hot climates where few clothes are worn) and in such circumstances modification of the questions is required (e.g. rise from a squat position).

List 5 Practical tips in performing pGALS

• Look for verbal and non-verbal clues of discomfort (e.g. facial expression, withdrawal)

• Do the full screen as extent of joint involvement may not be obvious from the history

• Look for asymmetry (e.g. muscle bulk, joint swelling, range of joint movement)

• Consider clinical patterns (e.g. non-benign hypermobility and Marfanoid habitus or skin elasticity) and association of leg length discrepancy and scoliosis)

pGALS starts with observing the child coming into the room, interaction with the parent or carer, and their interest in play or activities such as using pencils or crayons. The child should ideally be undressed but patience and opportunistic examination are needed as many CYP are reluctant to undress—prior request to bring along shorts and T-shirt and provision of privacy to change will facilitate the assessment. As a minimum, the child should be barefoot, the legs exposed to include the knee and thigh and arms to include the elbows. The torso can be exposed to assess the spine in due course.

Observation with the child standing should be done from the front, from behind the child, and from the side. The examination of the upper limbs and neck is best done with the child sitting on an examination couch facing the examiner. The child can copy the various manoeuvres as performed by the examiner. The child should then lie supine to allow the legs to be examined and then stand again for spine assessment. Throughout pGALS, the sequence of ‘look, feel, move’ is followed and checking for verbal and non-verbal signs of discomfort. It is important to check carefully for symmetry as the changes can be subtle (skin changes, joint swelling and deformity, muscle bulk, and ranges of joint movement). The features of inflammatory arthritis include joint swelling, warmth, loss of movement and tenderness on examination—an isolated painful swollen joint warrants mandatory investigation to exclude sepsis and it is noteworthy that a septic joint may not be hot or red. Conversely, in a well child with a monoarthritis, in the absence of trauma and sepsis, JIA is a likely diagnosis although in some geographical areas, mycobacterial infection or Lyme disease are common and need to be excluded.

From the front and back, leg alignment problems such as valgus and varus deformities can be observed and muscle bulk can be assessed. Scoliosis may be suggested by unequal shoulder height or asymmetrical skin creases on the trunk and may be more obvious on forward flexion. Subtle abnormalities at the ankle (such as swelling, valgus deformity) are often more obvious from behind the child. Leg length inequality may be more obvious from the side profile and suggested by a flexed posture at the knee; if found, then careful observation of the spine is important to exclude a secondary scoliosis.

Gait is assessed in the context of normal development (List 4 and Table 1). Inability to walk on heels or on tiptoe is a good screening manoeuvre for the ankle and foot, especially as foot or ankle involvement is common in JIA, and enthesitis is a feature of enthesitis-related arthritis. Sever’s disease (an osteochondritis of the calcaneum) may also present with a painful heel but the site of tenderness is distal to the enthesis.

The pGALS assessment of the arms examines several joints together with each composite movement. Hypermobility is suggested with increased flexion/extension of the wrists (hands palm to palm and hands back to back), increased extension of the elbows (arms outstretched) and the shoulders (“hands behind the neck”).

The pGALS assessment of the legs with the child supine includes observing for leg length (check that the pelvis is straight to avoid false positives), symmetry of muscle bulk (quadriceps wasting is common with JIA involving the knee), and alignment (looking for valgus or varus deformity). Lack of full extension of the knee is best observed when the legs are supine and the legs held extended; lack of passive hyperextension at the knee may be consistent with inflammatory joint disease (particularly if asymmetrical) and may remain as a physical sign with inactive arthritis.

Interpretation of pGALS in the context of inflammatory joint disease, is helped by knowledge of the characteristic patterns in JIA subtypes. For example, in a child with juvenile psoriatic arthritis, there may be asymmetrical joint involvement involving small and large joints, and this may include dactylitis or ‘sausage digit’ (due to arthritis and associated tenosynovitis). Isolated hip joint involvement is unusual as a presentation of JIA (with the exception of Enthesitis Related Arthritis), and other pathologies including orthopaedic conditions (Perthes, developmental dysplasia, slipped upper femoral epiphysis) and sepsis (including mycobacterial infection) need to be excluded. Referred pain such as from the hip or thigh as a cause of knee pain in the absence of physical signs at the knee must also be considered.

**Figure 2:**
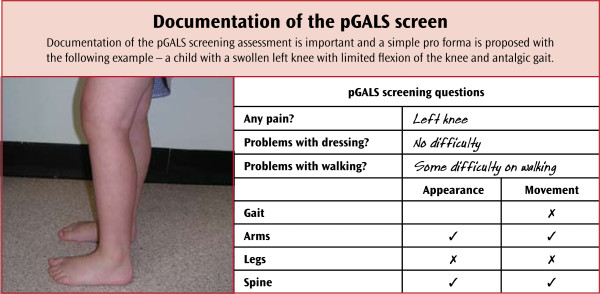
**Documentation of pGALS -(reproduced by kind permission of Arthritis Research UK (**http://www.arthritisresearchuk.org**) from: Foster HE, Jandial S. pGALS – A Screening Examination of the Musculoskeletal System in School-Aged Children.** Reports on the Rheumatic Diseases (Series 5), Hands On 15. Arthritis Research Campaign; 2008 June)

Documentation of pGALS within a standard medical clerking is important. A simple proforma is proposed (Figure 2).

### Paediatric Regional Examination of the Musculoskeletal System (pREMS)

Following pGALS, the observer is directed to a more detailed examination of the relevant area(s). A consensus approach to paediatric Regional Examination of the Musculoskeletal System (called pREMS) has been developed from observation of doctors and allied health professionals working in pMSK medicine [18]. pREMS is based on the ‘look, feel, move, function’ principle similar to that of adult REMS [19] for each joint or anatomical region, with active movements performed first and then passively by the examiner; the addition of ‘measure’ for some joints is included pending the clinical scenario (e.g. leg length discrepancy or thigh wasting). pREMS is primarily aimed at specialist pMSK training and as a minimum level of competence. pREMS is relevant to paediatric rheumatology although clearly many components are relevant to paediatric orthopaedics and allied health professionals.

### Teaching and learning of pGALS

We know that teaching of pMSK medicine is often absent in medical schools [7,20], and many doctors who are involved in the initial care pathway of CYP, lack the confidence and performance of pMSK clinical skills [3,6,8]. These observations may contribute to the observed delay in access to care that is reported in JIA [21-25] and other significant disease with MSK presentations [26,27].

We believe that pGALS is an essential clinical skill to be acquired as a minimum, by all medical students as part of undergraduate training and incorporated in the training of other clinicians (such as allied health professionals) as appropriate pending their clinical duties. We believe that learning of pGALS at medical school will equip all doctors to have the basic skills to triage CYP with MSK presentations, irrespective of their subsequent career path. To this end, consensus methodologies have derived a set of pMSK medicine learning outcomes for medical students [Jandial in preparation], which include pGALS and essential knowledge to aid assessment (including normal development, normal variants, red flags and common/significant pathologies to warrant concern). It is hoped that these pMSK learning outcomes will form the basis of a child health curriculum for medical schools (work in progress in UK). We envisage that pGALS is the basic clinical skill and pREMS, as a more advanced skill set, is directed at postgraduate medical training to promote refinement of pMSK clinical skills required by doctors working within pMSK medicine.

Challenges remain about the integration of pMSK teaching into medical school curricula; these include lack of confidence to teach MSK examination amongst paediatricians [Jandial, in preparation] and availability of pMSK specialists [20]. In our institution, performance of pGALS as a clinical skill and limping child as a key presentation (to cover essential knowledge) are mandatory learning outcomes for all medical students and taught by general paediatricians within child health; competence and knowledge are assessed by Objective Structured Clinical Examination (OSCE) and written examinations with high pass rates. Details of how teaching and learning of pGALS may be delivered is not covered in this article but is aided by all students having access to free educational resources that we have developed (http://www.arthritisresearchuk.org/health-professionals-and-students/video-resources/pgals.aspx) with further e-learning support in progress. Since the introduction of mandatory pMSK learning outcomes, we have noticed a marked increase in requests to attend paediatric rheumatology clinics as part of student learning and this has had a positive impact on interest in rheumatology as a career choice for several graduating doctors.

pGALS is included in many textbooks for undergraduate and postgraduate training as well as numerous e-learning modules for family medicine (http://www.e-lfh.org.uk/projects/general-practitioners/), general paediatricians and is a component of the mandatory paediatrics professional examinations (http://www.rcpch.ac.uk/training-examinations-professional-development/assessment-and-examinations/examinations/examinations).

pGALS may well have a role in low resource countries where it’s use may help allied health professionals to triage CYP; preliminary work has demonstrated the feasibility and practical use of pGALS in Malawi [16] and Peru [Abernethy, in preparation]. We are aware that pGALS is widely taught in many parts of the world and translations of pGALS into other languages to aid teaching and learning, as well as use in clinical practice, are in progress. Currently translations are available in Mandarin [Chan, in preparation] and Spanish [Abernethy, in preparation] with free resources to aid dissemination being prepared.

We believe that it is important that the paediatric rheumatology community continue to lobby for inclusion of pMSK teaching at their institutions, and embrace opportunities to support their paediatric colleagues to teach MSK clinical skills (i.e. “Teach the teachers”) to ensure that pMSK teaching is established in the basic clinical skill set acquired by all medical students. We believe that teaching of evidence and consensus based pMSK learning outcomes which include pGALS will be facilitated by the availability of free resources (see Further information). Such initiatives are likely to have many positive influences including increased awareness about pMSK problems, paediatric rheumatology as a speciality, and facilitate clinical skill acquisition, which will hopefully result in earlier recognition of significant MSK disease and access to care.

## Conclusions

pGALS is a simple, quick assessment of the musculoskeletal system and validated for use in the school aged child. It is useful to discern abnormal from normal joints and can detect abnormalities that may not be apparent from the history alone. The teaching and learning of pGALS as a basic clinical skill is an important step to raise awareness about the importance of joint examination and facilitate earlier diagnosis of rheumatic disease in children.

### Further information and reading

A full demonstration of pGALS and supportive documents are available as a web-based free resource: (http://www.arthritisresearchuk.org/health-professionals-and-students/video-resources/pgals.aspx). A demonstration of pREMS and supportive resources are in progress (to be launched early 2014).

## Competing interests

The authors declare that they have no competing interests.

## Authors’ contributions

HF compiled the manuscript. SJ contributed to the format of the article and commented on the manuscript. Both authors read and approved the final manuscript.
